# Prenatal Exposure to Gestational Nicotine before Neurulation is Detrimental to Neurodevelopment of Wistar Rats’ Offspring

**DOI:** 10.21315/mjms2018.25.5.4

**Published:** 2018-10-30

**Authors:** Gabriel Olaiya Omotoso, Risikat Eniola Kadir, Fatima A. Sulaimon, Rukayat Jaji-Sulaimon, Ismail Temitayo Gbadamosi

**Affiliations:** Department of Anatomy, Faculty of Basic Medical Sciences, College of Health Sciences, University of Ilorin, Ilorin, Nigeria

**Keywords:** morphology, nicotine, prefrontal cortex

## Abstract

**Background and aim:**

This study aimed to determine the effect of gestational nicotine exposure before neurodevelopment on the morphology and histology of the prefrontal cortex (PFC) in rats.

**Methodology:**

Adult female Wistar rats were time-mated and grouped into three categories: (a) control–given 0.1 mL of normal saline, (b) low-dose nicotine–given 6.88 mg/ kg/d/0.05 mL, and (c) high-dose nicotine–given 13.76 mg/kg/d/0.1 mL in two divided doses. Treatment was given intraperitoneally from gestational days 2 to 6. On postnatal day 15 (P15), the pups were separated from their mothers, anaesthetised and sacrificed, followed by intracardial perfusion with 4% paraformaldehyde. PFC was excised from the brain and processed for tissue histology, histochemistry, and morphology of brain cells.

**Results:**

Gestational nicotine exposure during the first week of gestation in rats significantly reduced birth weights in nicotine-treated groups compared with control; it, however, accelerated body weights, altered neuronal morphology, and elevated astrocytic count significantly, while oligodendroglial count was slightly increased in the PFC of juvenile rats examined at P15.

**Conclusion:**

These alterations revealed that gestational nicotine exposure before the commencement of the cellular processes involved in brain development negatively affects neurodevelopment, and this could result in neurological dysfunctions in later life.

## Introduction

Gestational exposure of the foetus to chemical injury is hazardous to human development beyond the prenatal stage (1). Neurodevelopment is susceptible to chemical insults, affecting cellular processes such as proliferation, migration, differentiation, synaptogenesis, myelination, and apoptosis (2). Exposure to neurotoxic substances during these periods contributes to a variety of neurodevelopmental and neurological disorders (3). The time of exposure and the type of substance are two important factors to consider when determining the vulnerability of the developing brain exposed to neurotoxicants (4).

Environmental contaminants hazardous to the development of the nervous system include lead, arsenic, organic mercury, polychlorinated biphenyls, organophosphates, and tobacco, amongst others (5, 6). Neurological disorders such as autism, attention-deficit disorder, mental retardation and certain psychiatric disorders have been associated with some of these chemical agents (7, 8). Tobacco itself comprises thousands of chemical constituents, many of which are toxic and carcinogenic to organs of the body (9). Nicotine is a major constituent in tobacco, most commonly consumed in the form of cigarette smoke (10). However, direct sources of nicotine include nicotine replacement therapy, which involves using nicotine skin patches or nicotine gum for smoking cessation (11, 12).

Nicotine is neuroteratogenic, and its activity is observable during prenatal and postnatal periods of neurodevelopment (13). Irrespective of the dose or amount exposed to nicotine produces substantial and long-lasting changes in the brain (14). Many studies have implicated nicotine in the causation of some neurological abnormalities in offspring of smoking mothers (15). Meanwhile, even second-hand exposure of pregnant women to tobacco smoke has been linked to increased risk of neurodevelopmental delay and adverse foetal outcomes, including preterm delivery and low birth weight (16).

The effect of nicotine on the central nervous system (CNS) is via activation of the nicotinic acetylcholine receptors (nAChRs) (17). According to Dwyer et al. (18), the expression of nAChRs begins in the second week of gestation in rodents, in a caudal to rostral pattern, and reaches the neocortex in the late third week of gestation.

The concentrations of nicotine in the foetal blood could be up to 15% higher than maternal levels (19). Chronic exposure leads to up-regulation of the nAChRs and increased receptor density of foetal and neonatal cerebral nAChRs. This stimulation results in premature onset of cell differentiation at the expense of replication, leading to cell death, structural changes in regional brain areas, and altered neurotransmitter systems (20).

Aberrant prenatal or postnatal nicotine exposure of foetal and neonatal brain to nicotine, through maternal smoking or nicotine replacement therapy (NRT), has detrimental effects on cholinergic modulation of brain development, leading to alterations in sexual differentiation of the brain, cell survival, and synaptogenesis (21). It promotes apoptosis and produces deficits in the number of neural cells and in synaptic function (22). The outcome of these effects could include deficits in later learning, memory, behaviour, and development (20).

Children prenatally exposed to tobacco smoke have more delinquent, aggressive and externalising behaviours, are more active and impulsive, and have more problems with peers (23). Most of these effects were found in human from exposure during the first trimester.

In rodents, however, which normally have 21 or 22 days of gestational period, there is a subtle difference (24). According to Rice and Barone (2), neurulation begins in the early part of the second week of gestation in rats, marking the onset of neurodevelopment. Specifically, neurogenesis in most cortical and subcortical regions of the brain starts at gestational day 9.5 and is completed by postnatal day 15 (25). This study hence aimed at determining what effects gestational nicotine exposure before the onset of neurodevelopment have on the prefrontal cortex of juvenile Wistar rats.

## Materials and Methods

### Animal Care

Handling and use of experimental animals were in accordance with the guidelines of the Institutional Animal Care and Use Committee and approved by the Postgraduate School, University of Ilorin, Nigeria.

Fifteen adult female Wistar rats with an average weight of 164.67 g were put in different cages (five in each cage) and housed in the Animal House of the Faculty of Basic Medical Sciences, University of Ilorin, at normal room temperature, and maintained under hygienic condition. They were fed on pelletised growers feed from United African Company Vital Feeds^®^ (Nigeria), containing 15% crude protein, 7% fat, 10% crude fibre, 1% calcium, 0.35% phosphorus, 2,550 kcal/kg of metabolised energy, as stated in the pack. Clean drinking water was provided ad libitum. The rats were apparently normal with no obvious abnormal characteristics prior to the onset of the studies.

### Determination of Oestrus Phase, Mating and Confirmation

The oestrus phases of the female rats were determined using the vaginal smear technique as described by Marcondes et al. (24). Mature male Wistar rats were introduced to the female rats overnight and in their proestrus phase. The presence of spermatozoa in the vagina, as examined from vaginal smear obtained the following morning and viewed under the light microscope, was indicative of successful mating (24); this was taken as day 0 of pregnancy (26).

### Experimental Design

The pregnant rats were randomly grouped into three categories: (a) control–received 0.1 mL of normal saline; (b) received low-dose nicotine (LDN; 6.88 mg/kg/d/0.05 mL); and (c) received high-dose nicotine (HDN; 13.76 mg/ kg/d/0.1 mL) in two divided doses (about 10 h interval between each dose) (26, 27). Nicotine (95%) was obtained from British Drug Houses Chemical Ltd. Poole, England. Administration of nicotine was intraperitoneal, with the aid of an insulin syringe (calibrated from 0.1–1.0 mL) for five consecutive days during the first gestational week from days 2–6. The pregnant rats were handled with utmost care during the experiment. After delivery, the weights of the pups were taken on the day of delivery and on postnatal day 15, just before sacrifice, with each group having about three to six pups.

### Animal Sacrifice and Tissue Perfusion Fixation

On postnatal day 15, the rats were intramuscularly anaesthetised with ketamine and transcardially perfused with normal saline to flush out the blood in the vessels, followed by 4% paraformaldehyde (PFA) in 0.1 M phosphate buffer to fix the brain tissue in situ. After perfusion, the skull was dissected to remove the brain; about 3 mm of the frontal lobe beginning from the frontal pole was excised, weighed and post-fixed in 4% PFA.

### Tissue Processing for Paraformaldehyde-fixed Tissues

Tissue processing was carried out using an automatic tissue processor (Leica TP1020, Leica Biosystems^®^, Germany) and a tissue embedding system (Leica EG 1160, Leica Biosystems^®^, Germany). Tissue blocks were sectioned using a rotary microtome (Leitz Wetzlar^®^ 1512, Germany) at a thickness of 5 μm. Staining of mounted tissue sections was carried out using Haematoxylin and Eosin staining technique (28) to assess the general microarchitecture of the prefrontal cortex; cresyl fast violet staining technique (29) was used for Nissl staining.

Tissue sections prepared for Nissl staining were used for cell count, using ImageJ–win32 computer software (National Institute of Health, USA). Counting of cells was carried out using a counter window of 1,024 × 768 pixels dimensions (resolution); width: 1,024 pixels (160 microns); height: 768 pixels (120 microns). Five different counter windows were analysed per slide and captured at different fields of the sections. The average of the five windows was determined and reported as number of cells per 160 × 120 μm slide area (or per counter window) (26). Neurons and glia (oligodendrocytes and astrocytes) were counted. The diameter of cell bodies and nuclei of neurons were also measured using Motic Images Plus 2.0 ML software (Motic China Group^®^, China).

### Data Analysis

The data obtained were subjected to statistical analysis using the GraphPad Prism^®^ software and were plotted using ANOVA with Tukey’s multiple comparisons test. Data obtained were presented as mean ± standard deviation, with determination of level of significance at *P*-values less than 0.05.

## Results

### Physical Observation

Observation of the pups in all the groups after delivery until postnatal day 15 did not reveal any obvious abnormality and no significant impairment in their activities such as movement and feeding.

### Birth Weight of Offspring Exposed to Nicotine during Pregnancy

**Figure 1:**
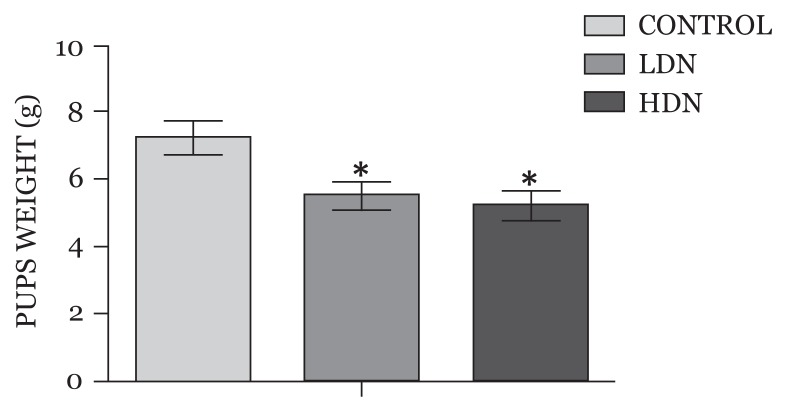
Birth weights of pups (mean, SD). Treatment groups had significant reduction in weights compared with Control *(*P* < 0.05) LDN: lower dose nicotine; HDN: higher dose nicotine

Reduction in birth weight occurred in both nicotine-treated groups compared with control (control: 6.96 ± 0.59, LDN: 5.50 ± 0.44), with rats exposed to the higher dose (HDN: 5.12 ± 0.36) of prenatal nicotine having the least birth weight; the weight difference between the two treated groups was statistically significant (*P* < 0.05) (Figure 1).

### Body Weight of Offspring on Postnatal Day 15

**Figure 2:**
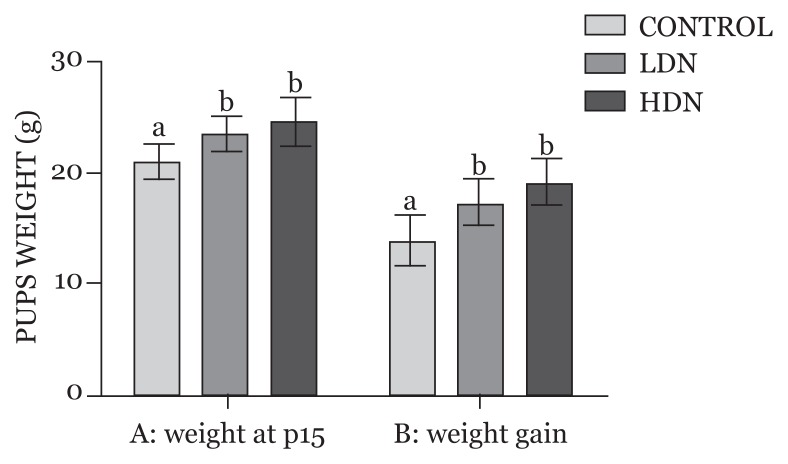
A: Body weights (mean, SD) of pups at postnatal day 15 (P15), and B: Weight gain between birth and P15. Mean values with different letters (a, b) signify statistically significant difference at *P* < 0.05 LDN: lower dose nicotine; HDN: higher dose nicotine

Body weights of rats at postnatal day 15 (P15) increased in nicotine-exposed groups, with animals exposed to higher dose of prenatal nicotine having the highest rate of weight gain. The differences in weight changes between the control (20.85 ± 0.35) and the treatment groups (LDN: 23.60 ± 0.63, HDN: 24.52 ± 1.17) were statistically significant (*P* < 0.05), while the difference between the low-dose nicotine (LDN) and high-dose nicotine (HDN) groups was not statistically significant (*P* > 0.05), even though the latter had a higher weight increase (Figure 2).

### Cell Count and Morphology

#### Neuronal count

**Figure 3:**
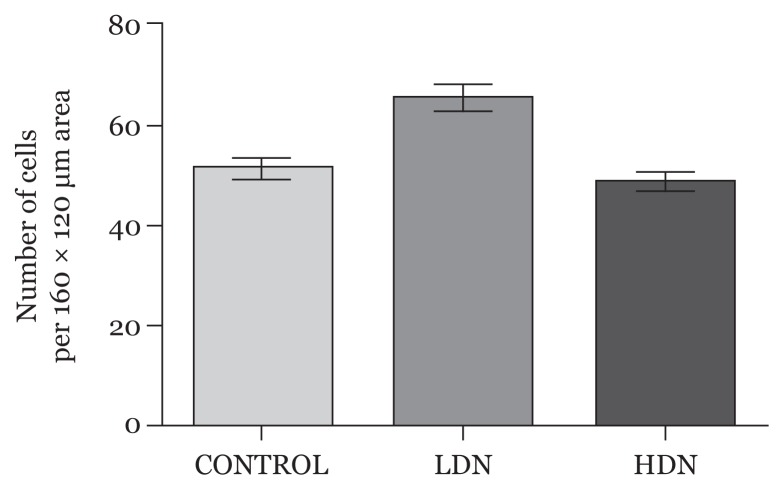
Average neuronal count per 160 × 120 μm area of the prefrontal cortex (mean, SD). Differences among the groups were not statistically significant (*P* > 0.05) LDN: lower dose nicotine; HDN: higher dose nicotine

Neuronal count revealed only a slight reduction in cell count in the HDN group compared with control, while the count markedly increased in the LDN group (*P* > 0.05) (Figure 3).

#### Oligodendroglial count

**Figure 4:**
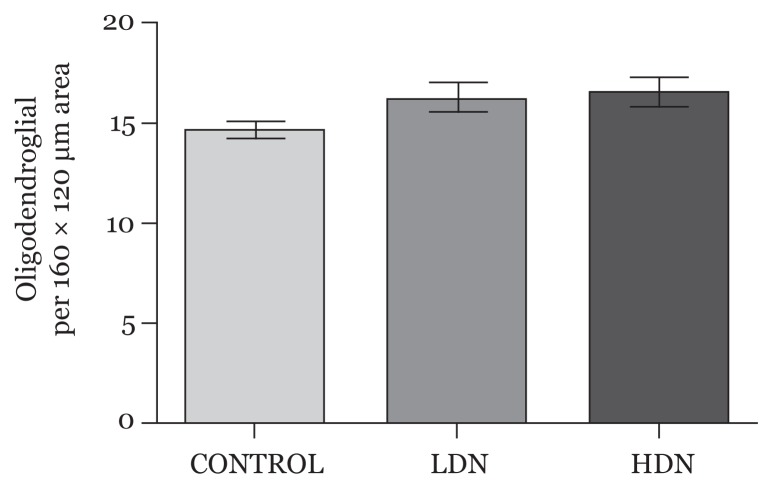
Average oligodendroglial count per 160 × 120 μm area of the prefrontal cortex (mean, SD). No statistically significant difference between the groups (*P* > 0.05) LDN: lower dose nicotine; HDN: higher dose nicotine

Increase in oligodendroglial count was observed in rats exposed to nicotine, with those treated with high-dose nicotine having a slightly higher count compared with the LDN group (*P* > 0.05) (Figure 4).

#### Astrocytic count

**Figure 5:**
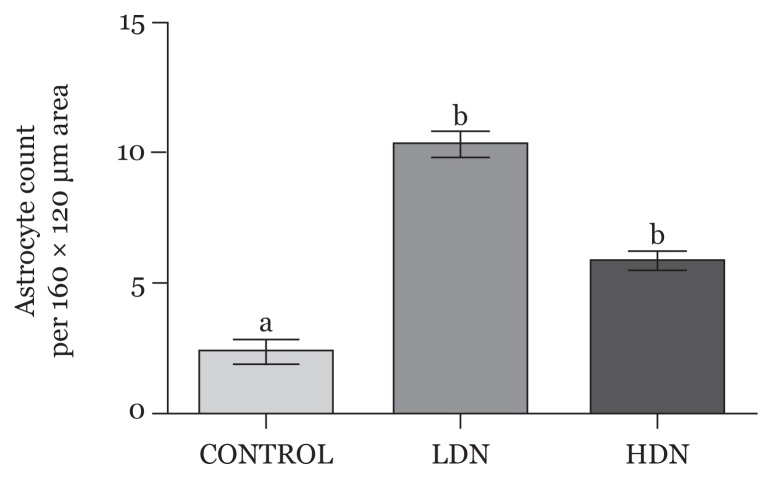
Average astrocytic count per 160 × 120 μm area of the prefrontal cortex (mean, SD). Bars with different superscript letters (a, b) in each group are statistically significant (*P* < 0.05) LDN: lower dose nicotine; HDN: higher dose nicotine

Astrocytic count significantly increased in both the lower-dose and higher-dose groups compared with control, and these changes were statistically significant (*P* < 0.05) (Figure 5).

#### Degenerated neuronal cells

Neuronal cells with altered structural morphology were also assessed and counted, and the percentage of such degenerated neurons was noted. The occurrence of degenerating neuronal cells was seen in both the nicotine-exposed groups in a dose-dependent pattern, with the high-dose group having more degenerated neurons compared with the low-dose group (Table 1).

#### Neuronal somatic diameter

**Figure 6:**
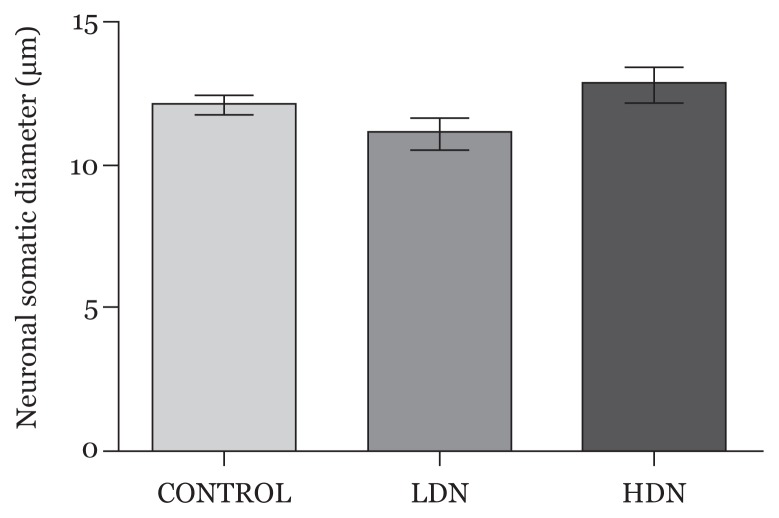
Diameters of cell bodies of neurons in the prefrontal cortex at postnatal day 15 (mean ± SD); differences were not statistically significant LDN: lower dose nicotine; HDN: higher dose nicotine

The size of the cell bodies of neurons in the PFC was reduced in the group exposed to LDN but increased in those exposed to HDN, though not significantly (*P* > 0.05) (Figure 6).

#### Nuclear diameter

**Figure 7:**
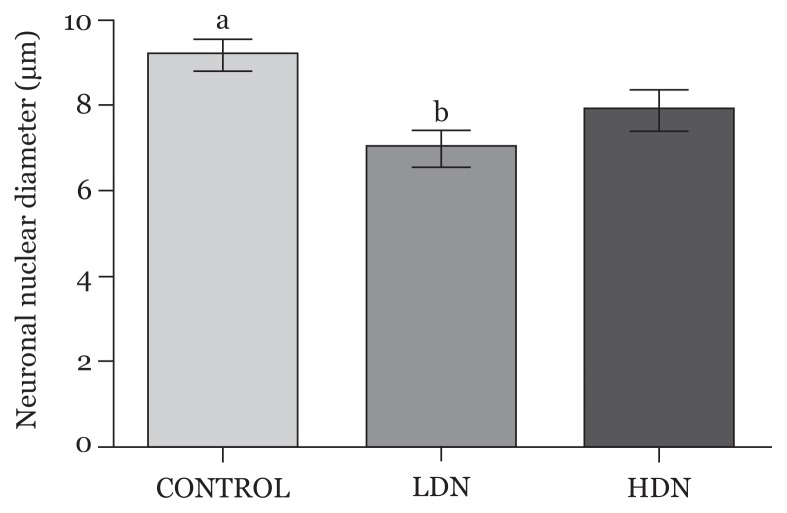
Diameters of nuclei of neurons in the prefrontal cortex (mean, SD). The difference between the Control (a) and LDN (b) were statistically significant (*P* < 0.05) LDN: lower dose nicotine; HDN: higher dose nicotine

There was reduction in nuclear diameter across the treatment groups, which was marked in the LDN group (*P* < 0.05) compared with control (Figure 7).

#### Nuclear-Somatic ratio (NSR)

**Figure 8:**
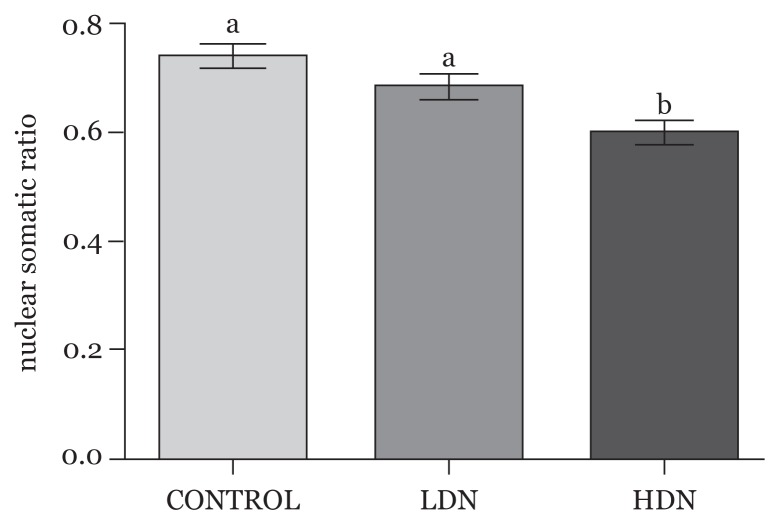
Nuclear-Somatic Ratio, as an index of nuclear size relative to the size of the neuronal cell body. Statistically significant differences were observed between Control (a) and HDN (b), as well as LDN (a) and HDN (b) *P* < 0.05, while difference in the Control (a) and LDN (a) was not significant (*P* > 0.05) LDN: lower dose nicotine; HDN: higher dose nicotine

The diameter of the nuclei of neurons relative to that of the cell bodies (nuclear-to-soma ratio) was measured (as an index of nuclear size). This was subsequently reduced in both treatment groups when compared with control, with statistically significant difference in the high-dose group and also between the two nicotine groups (*P* < 0.05) (Figure 8).

#### Perinuclear-Somatic space (PSS)

**Figure 9:**
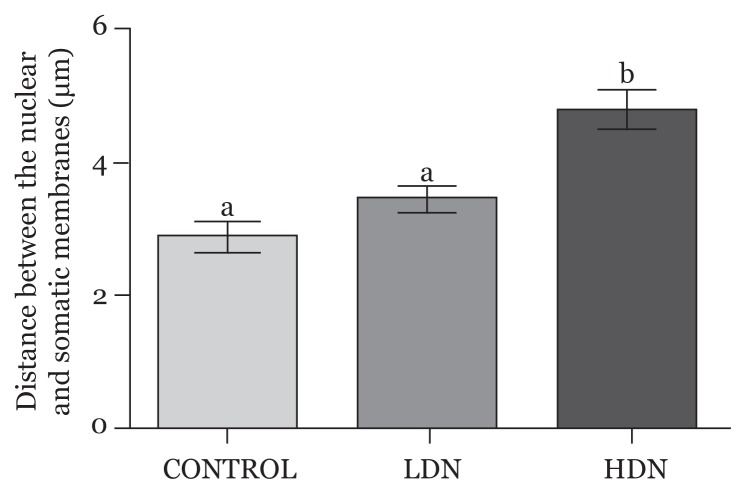
Perinuclear-Somatic Space (space between the nuclear membrane and somatic membrane) of neurons in the prefrontal cortex. Statistically significant differences were observed between Control (a) and HDN (b), as well as LDN (a) and HDN (b) *P* < 0.05, while difference in the Control (a) and LDN (a) was not significant (*P* > 0.05) LDN: lower dose nicotine; HDN: higher dose nicotine

The space between the nuclear envelope and the somatic membrane, occupied by the cytoplasm, was measured. This space was increased in all the treatment groups, more in the HDN (*P* < 0.05) compared with control and the LDN group (*P* < 0.05) (Figure 9).

#### Histology of prefrontal cortex (PFC)

**Figures 10 (A–F):**
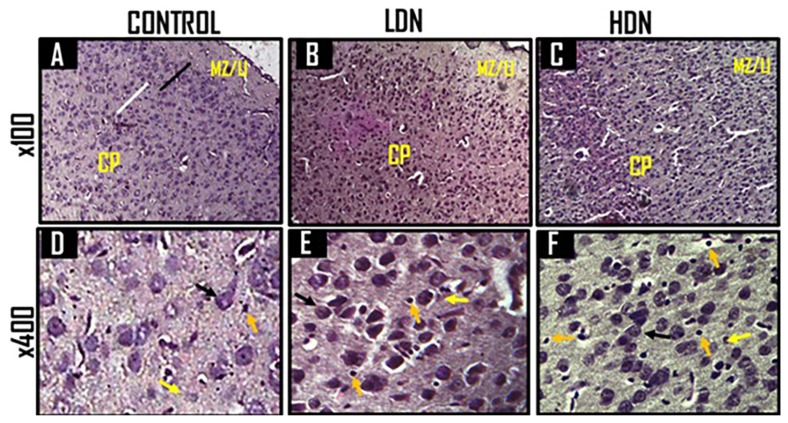
Representative photomicrographs of Haematoxylin & Eosin staining of the prefrontal cortex (PFC) of 15 days old Wistar rats treated during the first gestational week with 0.1 mL normal saline (A&D: Control); lower dose nicotine (B&E: 6.88 mg/kg/d LDN); and higher dose nicotine (C&F: 13.76 mg/kg/d HDN). The higher magnifications (D, E and F) captured the cortical layer V Figure 10A showed fairly delineated cortical layers into marginal zone (MZ) or Layer I (LI) and cortical plate (CP). The different layers of the CP were not distinctively delineated, though Layers II/III (black line) and Layer IV (white line) can fairly be seen. In Figure 10B, LI and LII/III can fairly be distinguished from the remaining deeper layers, and both appeared wider than those of Figure 10A. Brain cells appeared smaller, more numerous and deeper stained than in Figure 10A. Figure 10C showed deeper stained cells compared with Figure 10A, but distorted and irregular architecture. The cortical layers were also poorly delineated. Figure 10D showed fairly well-delineated cellular architecture with a few cells with prominent nucleoli. Neurons (black arrows) in Figure 10E appeared more deeply stained with more astrocytes (yellow arrows) and oligodendrocytes (orange arrows) compared with Figure 10D. There were many degenerated neurons (black arrows) in Figure 10F and the oligodendrocytes (orange arrows) were more than in Figures 10D and 10E.

**Figures 11 (A–F):**
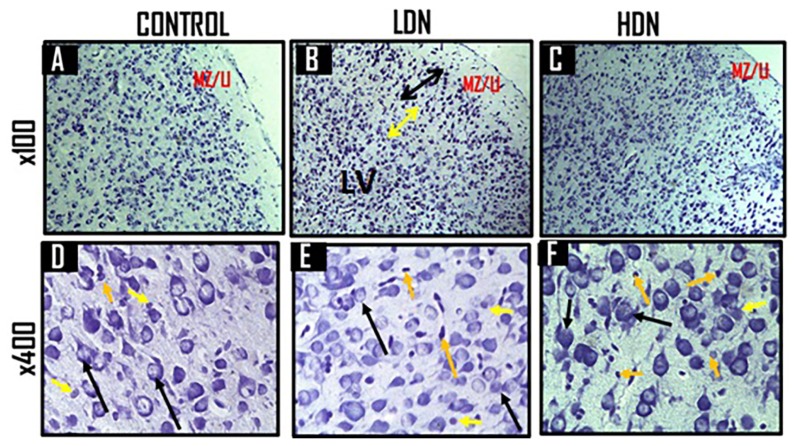
Representative photomicrographs of cresyl fast violet staining of the PFC of 15 days old Wistar rats treated during the first gestational week with 0.1 mL normal saline (A&D: Control), 6.88 mg/kg/d nicotine (B&E), and 13.76 mg/kg/d nicotine (C&F). The higher magnifications (D, E and F) captured the cortical layer V Figure 11A revealed fairly stained section of the PFC with many neurons and oligodendrocytes. The cortical plate was well defined from the marginal zone (MZ), but not well delineated into different cortical layers, unlike in Figure 11B, and was positive for Nissl staining (Figure 11B). There was a slight increase in the thickness of the MZ [or Layer I (LI)], with reduced cell sizes; Layers II and III (black line) had deeply stained cell bodies with many small pyramidal cells; Layer IV (yellow line) was lightly stained compared with other gray cortical layers, while Layer V (LV) contained largely pyramidal cells, and less deeply stained compared to LII/III. Figure 11C had fewer and slightly large-sized neurons compared with Figure 11B, but less well delineated CP. Figure 11D showed neurons (black arrows) with prominent nuclei, nucleoli and myelinated axons, and glial cells such as oligodendrocytes (orange arrows) and few astrocytes (yellow arrows). Figure 11E showed increased population of neurons (black arrows) with prominent nuclei and nucleoli, but reduced sizes (compared with Figures 11D and 11F), and increased number of oligodendrocytes (orange arrows) and astrocytes (yellow arrows) compared with (Figure 11D). Figure 11F showed more positivity for Nissl staining, but had many degenerated neurons (black arrows) with less prominent nucleoli, many reactive astrocytes (short yellow arrows) and many myelinating oligodendrocytes (short orange arrows).

The histology of the PFC of animals administered with normal saline (control) in the first week of gestation revealed apparently normal architecture, fairly delineated cortical layers, divided broadly into the marginal zone (MZ) and cortical plate (CP), with numerous cells (Figure 10A) and good Nissl staining of the neurons (Figures 11A and D). There were many well-formed neurons and oligodendrocytes but few astrocytes. Some of the neurons had prominent nucleoli (Figures 10A, 10D, 11A, 11D).

In animals exposed to low-dose nicotine (Figures 10B, 10E, 11B, 11E), the prefrontal cortical layers (Layers I to V) were better delineated compared with control (Figure 11A) and the high-dose nicotine group (Figure 11C). The layers were distinguishable by the staining intensity of the cells. Layer I, or marginal zone, was predominantly white matter with few cell bodies present; the cortical thickness was increased compared with that of control. There were more cell bodies, with reduced sizes, but were more deeply stained. Cortical Layers II and III (LII/III) were made up of more deeply stained cell bodies and many small pyramidal cells; the proximal parts of the axons were visible. Layer IV was lightly stained compared with the other layers of the cortical gray matter (cortical plate). Layer V was made up of few large pyramidal cells and less deeply stained compared with LII/III (Figure 11B). LI and LII/ III appeared wider in thickness compared with those of control, and the cell bodies generally showed positivity to Nissl staining. The number of glial cells (oligodendrocytes and astrocytes) increased, particularly in their gray matter (Figure 10E), and the ratio of oligodendrocytes to astrocytes was markedly reduced.

The HDN-treated rats showed considerable disruption of the microarchitecture and deep tissue staining (Figure 10C, 10F, 11C, 11F). The cortical layers were poorly delineated, while some of the neurons demonstrated were degenerated (Figure 10F). There was reduction in the thickness of Layers I to III, whereas the diameter of Layer IV was increased. Layer II/III was lightly stained, while Layer V was less deeply stained and poorly delineated from Layer IV. The number of neurons was reduced and their nuclei appeared smaller in size, and the nucleoli were less distinct (Figure 11F). There were more obvious degenerated neurons as shown by nuclear disintegration, membrane damage, and presence of many reactive astrocytes near the degenerating neurons. A minimal increase in the number of oligodendrocytes was observed compared with control (Figure 11D) and the LDN group (Figure 11D). The Nissl bodies of the neurons were positively stained.

## Discussion

### Gestational Nicotine Exposure is Associated with Low Birth Weight but Accelerated Gain in Body Weight in Wistar Rats’ Offspring

Previous studies have reported that low birth weight usually accompanies prenatal exposure to nicotine-containing substances, such as tobacco or cigarette smoke (30). In the current work, reduction in birth weight occurred in pups exposed to intraperitoneal nicotine, irrespective of the dose, during the first week of gestation, with the rats exposed to higher concentration of nicotine and having the lowest birth weight. One of the mechanisms through which nicotine affects foetal growth is via its effect as a vasoconstrictor. Nicotine causes uterine vascular constriction with a resultant reduction in perfusion of foetal tissues, and this can also lead to intrauterine growth restriction (31). Hence, there is reduction in the delivery of oxygen and nutrients to the foetus, which can contribute to intrauterine growth retardation and eventual low birth weight when the baby is born. These events, to some extent, explained the reason for the reduction in birth weight seen in the current study.

One important reason for smoking initiation in young people, especially teenage girls, is the ability of nicotine to regulate appetite and body weight and, once initiated, the addictive property of nicotine makes quitting difficult (32). In the pregnancy state, these effects pose a major challenge to the developing foetus (12, 20). Nicotine increases the serum levels of hormones, such as norepinephrine, dopamine, and serotonin, which are capable of suppressing appetite and enhancing weight loss (33). An extension of these hormonal effects could be contributory to why prenatal nicotine administration causes intra-uterine growth retardation and the eventual low birth weight (12, 20, 34). Use of tobacco during pregnancy has been associated with a higher rate of pregnancy complications with significant implication on both maternal and foetal outcomes (35). The appetite-suppressing effect of nicotine is on the mother rats, which could result in intrauterine growth restriction, involving multiple foetal organs (36).

The increase in body weight that characterised the early postnatal life of prenatally exposed rats as assessed at P15 in this study can be attributed to nicotine exposure during the early gestational period and not necessarily an exposure throughout the whole gestational period. As demonstrated by Fornari and colleagues (37), nicotine cessation is associated with increased hypothalamic neuropeptide Y and Agouti-related protein (AgRP), a consequent increased drive to eat and reduced capacity for energy expenditure. This could account for the higher rate of weight gain observed after birth in the nicotine-exposed rats.

Furthermore, prenatal nicotine exposure has been shown to cause glucose intolerance and impaired brain response to insulin in the offspring (38). Studies on adult rats at week 26 revealed endocrine and metabolic changes in prenatally exposed rats that are consistent with glucose metabolism disturbance, which could result in type 2 diabetes (39). According to Chen et al. (34), children prenatally exposed to nicotine could experience a ‘catch-up growth’, with subsequent childhood obesity. Alterations in the central endocrine control of body weight homeostasis could be responsible for the increased adiposity or increased body weight associated with gestational nicotine exposure, since the signals for body weight regulation and energy balance are ultimately integrated in the hypothalamus (12). These changes result in increased appetite in offspring, similar to the observation following nicotine cessation.

### Cytoarchitectural Changes and Neuronal Damage is Associated with Gestational Nicotine Exposure

Nicotine administration affects the neuronal cells. Exposure of rats to low-dose nicotine in the first week of gestation was associated with increased neuronal count, unlike as previously shown (40, 41). However, the neuronal cells were smaller and their nuclei were also reduced in size. This showed that, although neuronal count increased, there was still impairment in the developmental processes; hence the neurons were not normal structurally and perhaps functionally. Higher nicotine concentration exposure caused reduction in cell counts alongside other degenerative changes, such as reduction in neuronal and nuclear sizes, nuclear disintegration, and compromise of membrane integrity. Some of these effects could be due to some delay in cell growth and affectation of cell division (40, 41).

Activation of nAChRs adversely affects brain morphogenesis, spontaneous neural activity, and neuronal survival in rodents; it also causes persistent neurochemical alterations (21). Neurodegenerative changes observed in the control rats could be due to ongoing apoptosis (programmed cell death), which is part of the normal processes involved in cell growth and development. At the time of sacrificing these rats at P15, neurodevelopment was still ongoing, and apoptosis was yet to cease (2). There could also be a possibility of enhanced or exaggerated apoptotic processes in the PFC of those rats exposed to nicotine prior to neurulation. Due to the minimal neuronal changes observed in the PFC of rats exposed to nicotine in the first week of gestation, the severity of resulting neurological damage might not be as overwhelming as those seen following exposure at other periods of gestation. Even though neurogenesis in rodents does not commence until the second week of gestation, exposure to nicotine prior to its onset could still affect the quality of neurons produced during the process of neurodevelopment.

The distribution of Nissl granules is a reflection of the protein synthetic activity of the neuron. Loss of Nissl granules or damage to the structure is an indication of alterations in neuronal protein synthesis (42). The presence of Nissl granules did not appear to be adversely affected in neurons of rats exposed to nicotine. This could be because gestational nicotine exposure at this time might not have severely affected the composition of the rough endoplasmic reticulum in the cytoplasm compared with the other groups. Furthermore, intact neurons were present in the PFC of rats exposed to nicotine during the first gestational week (GW), resulting in a better preserved cytoplasmic content.

### Gestational Nicotine Exposure before Gliogenesis has Differential Effects on Both Oligodendrocyte and Astrocyte Counts

Oligodendrogenesis, a component of gliogenesis, begins a few days after neurulation (2). This neurodevelopmental process had not commenced when the rats were treated with nicotine in utero. Our current study showed that gestational nicotine exposure at this period did not have any significant effect on the population of oligodendrocytes in the PFC examined at P15 other than a slight increase in cell count.

We have previously demonstrated that prenatal nicotine exposure in the second and third weeks of gestation in rats was associated with reduced oligodendrocyte count and dysmyelination of axons in rats examined on postnatal day 15 (43). The implication of this is that gestational nicotine exposure prior to the commencement of oligodendrogenesis may not affect oligodendrocyte precursor cells from where they are produced; hence their functions especially in myelin production would be preserved.

However, unlike oligodendrocytes population, astrocyte count increased in the PFC of juvenile rats exposed to nicotine. This could be due to a compensatory increase in glial cells or a reactive mechanism of astrocytes against an ongoing cellular degeneration induced by gestational nicotine exposure. Although the time of exposure was before the commencement of gliogenesis (2) and a direct effect on astrocyte development could not be ascertained, the observation of this study corroborates previous studies that showed the defensive role of astrocytes in brain pathology (44). Astrocytes are critical for the control of brain homeostasis and the intrinsic brain defense system (45). Injury to the brain results in astroglial defense response called reactive astrogliosis, which is essential for both limiting the areas of damage by scar formation and for the post-insult remodeling and recovery of neural function (45). Reactive astrogliosis is a nonspecific but highly characteristic response that involves various morphological and molecular changes (46).

How astrogliosis occurs is not well understood; however, damaged neurons could induce astrogliosis, and the latter has been used as an index for underlying neuronal damage (44). According to Sun and Jakobs (46), the glial scar produced by reactive astrocytes could impede axon regeneration.

## Conclusion

Gestational exposure to nicotine during the first week of gestation of rats causes significant alteration in birth and body weights and alters neuronal morphology and astrocytic count in the prefrontal cortex of juvenile rats in the postnatal life. These developmental changes could adversely affect the functions of the prefrontal cortex with possible features resembling those of children exposed to prenatal nicotine.

## Figures and Tables

**Table 1:** Proportion of normal neurons and degenerated neurons per Counter Window in layer V of the lateral prefrontal cortex of rats presented as mean ± SD and percent

Neurons	First Gestational Week		

	A	B	C
Average total neuronal count	48.8 ± 3.7	67.6 ± 10.0	46.8 ± 5.0
Degenerated neurons (%)	12.3	35.8	53.0
Normal neurons (%)	87.7	64.2	47.0
